# Validation of the Psychopathic Processing and Personality Assessment in the Czech Republic

**DOI:** 10.3389/fpsyt.2026.1694620

**Published:** 2026-02-20

**Authors:** Ivan Sebalo, Martina Sebalo Vňuková, Martin Anders

**Affiliations:** Department of Psychiatry, First Faculty of Medicine Charles University and General Teaching Hospital, Prague, Czechia

**Keywords:** cross-cultural validation, psychopathic processing and personality assessment (PAPA), psychopathy traits, sex-specific structures, three-factor solution

## Abstract

**Objective:**

This study aims to validate the Czech-language Psychopathic Processing and Personality Assessment (PAPA) by examining its factor structure and psychometric properties in a non-forensic community sample in the Czech Republic.

**Methods:**

The study included 1013 adult participants (51.73% male, 48.27% female) with an average age of 42.14 years (SD = 8), drawn from the general population of Czech parents with at least one child. Participants completed the PAPA, a 29-item self-report questionnaire. The sample was divided into two groups for exploratory (n=304) and confirmatory factor analysis (n=709). Data were analysed using Principal Component Analysis (PCA) and Confirmatory Factor Analysis (CFA) to identify and validate the factor structure.

**Results:**

The exploratory analysis suggested a three-factor solution comprising hostile attitudes, manipulativeness, and callous-unemotional traits, which was confirmed through CFA. The model demonstrated acceptable fit indices (CFI, RMSEA, SRMR), indicating a robust structure for both male and female participants. Internal consistency for the factors ranged from acceptable to good.

**Conclusions:**

The validation of PAPA in Czech revealed a three-factor structure consistent with core psychopathy traits. This tool provides a reliable measure for assessing psychopathy in the Czech general population, highlighting cultural and sex-specific nuances in psychopathy assessment. Future research should explore the inclusion of the boldness dimension and validate the tool in forensic settings.

## Introduction

Psychopathy, or psychopathic personality disorder, comprises a variety of personality traits and behaviours. Conceptually, according to the Triarchic Model of Psychopathy (TriPM), psychopathy can be understood as the interaction of *disinhibition*, defined as externalizing behaviour and low impulse control, either with *meanness* (a lack of empathy, excitement seeking, and the use of cruelty for self-empowerment) or *boldness* (the ability to stay calm and maintain focus in stressful or threatening situations) (1). Moreover, Hare and Neumann (2) posit that psychopathy has four major components, which align with four facets of the Psychopathy Checklist-Revised (PCL-R) (3). These four components include affective traits (e.g., lack of guilt); interpersonal traits (e.g., pathological lying); lifestyle characteristics (e.g., impulsivity); and antisocial behaviour (e.g., criminal versatility). Finally, an individual who, at the minimum, is an unreliable member of the community and, at the maximum, a threat to others is considered to meet the definition of psychopathy. Since it comprises a cluster of personality traits, psychopathy is suggested to occur on a continuum. Unsurprisingly, Hare (3) places psychopathy among the most important concepts within forensic fields, as individuals with this disorder require high-intensity and time-consuming treatment and management programs.

Indeed, while the prevalence of psychopathy as assessed via the PCL-R or earlier versions (for an overview, see Hare et al. (4)) is 1.2% in the general population (5), that among offenders charged with homicide crimes ranges from 27.8% to 34.5% (6). Moreover, even when other measures of psychopathy (e.g., the Levenson Self-Report Psychopathy Scale (LSRP) (7)) are taken into account, the average prevalence in the community is 4.5% (5). This clearly shows the disproportionate strain that individuals with psychopathy place on society via the harm that they inflict and the resources devoted to their rehabilitation. Similarly, using a nationally representative sample of American adolescents, Beaver et al. (8) showed that even when a more personality-focused instrument was used to assess psychopathy (as opposed to the PCL-R, which posits criminal behaviours as essential for diagnosis), the presence of these traits was significantly associated with increased chances of being arrested, being incarcerated, and having self-reported delinquency. However, instruments that encompass more constructs and risk factors are poorer predictors of the risk of violent behaviour than the presence of psychopathy and scores determined via instruments that assess psychopathy (9, 10).

Nevertheless, psychopathy is associated with severe and impactful criminal conduct and, owing to its features, often indicates the need for challenging treatment and rehabilitation. Although the previously held view that patients with this condition cannot be treated in the sense of reducing the risk of recidivism is inaccurate (10, 11), considerable resources are still required. Furthermore, because they are often high-risk offenders, individuals with psychopathy require a focus primarily on dynamic risk factors and high intensity, with the potential for a reduction in recidivism rather than the complete certainty of rehabilitation (12). Furthermore, there is a debate about the accuracy of the conceptualisation of psychopathy, which translates into the debate on the utility of existing measures for assessing psychopathy and culminates in the discussion of whether specific treatment programs that differ from those offered to high-risk patients are needed for individuals with psychopathy (13). Taken together, these findings indicate that while the relevance of psychopathy within forensic treatment is not contested, there is substantial disagreement regarding the exact symptoms that constitute psychopathy. Consequently, identifying or establishing an effective treatment requires considerable effort.

The first step to overcoming the current issues associated with the management of psychopathy is establishing its definition, which is inseparable from the instruments currently used to assess it. As noted above, the PCL-R and its predecessors have long been considered the gold standards for assessing psychopathy in forensic settings and, as such, are the most widely used measures (14, 15). Accordingly, psychopathy comprises two primary factors: interpersonal-affective traits, which include the aforementioned affective aspects and interpersonal traits, and antisocial lifestyle characteristics, which incorporate corresponding behaviours (16). Within this conceptualisation, psychopathy is considered a dimensional construct for which an individual must reach a threshold score of 30 (out of a maximum score of 40) points concerning their behaviours and personality traits to receive such a diagnosis. An alternative, but still dimensional, three-factor model with a clear distinction among interpersonal, affective, and behavioural factors was proposed by Cooke and Michie (17). However, the exclusion of specific antisocial factors has been argued against (2). Furthermore, the four-factor (sometimes called facet) structure underpinning two more significant factors was shown to have a good fit (18) in an offender sample.

Nevertheless, the PCL-R conceptualisation has come under further scrutiny. First, owing to the entanglement of psychopathy with societal norms, the validity of the application of an instrument designed based on male Canadian offenders for female offenders is questionable (19). Although the PCL-R still has acceptable predictive validity for female offenders, essential sex differences are still present, including lower overall scores, less physical violence, and higher promiscuity scores, which can be tied to sex biases in assessors (20). The current, ever-growing body of literature suggests that while there are some shared presentations of psychopathy between the sexes, differences are still present and correspond to differences in base rates. For example, women are reported to have lower rates of previous conduct problems and violent recidivism but higher levels of emotional dysregulation and sexual promiscuity or risk-taking (for a complete overview, see Verona and Vitale (21)). This, in turn, raises the need to either refine the overall conceptualisation and, as a consequence, assess the available tools considering the specifics of female psychopathy, or to reconstruct the tools entirely.

The second point of criticism of the PCL-R is theoretical in nature. Including criminal behaviour as a symptom of psychopathy increases the risk of circular argumentation, where criminal behaviour is explained by psychopathy, which already includes criminal behaviour as a defining characteristic. Furthermore, the PCL-R arguably lacks items that capture the *boldness* dimension of psychopathy within the Triarchic Model (22). This not only raises the yet unanswered question of whether the PCL-R fails to capture a relevant aspect of psychopathy, captured by other measures such as the Triarchic Measure of Psychopathy^1^ (23, 24), but also suggests that this instrument does not assess the aspect of psychopathy that has the most potential to yield adaptive results (12).

The last umbrella of challenges is related to the utility of the PCL-R. While this scale is the most widely used measure in forensic contexts, legal experts and researchers have expressed concerns about its use (10, 14). The most highlighted issue is the lack of association between the PCL-R score and treatment outcomes or violent conduct during or after incarceration. Furthermore, in research, the PCL-R was developed using forensic samples, and its administration requires collateral information and clinical judgment when scoring each item. In other words, this scale is not a self-reported measure that can be used easily in research with community participants. This, in turn, limits the scope of psychopathy research; the restriction of studies to the forensic population means potential oversight of the constructs relevant for individuals who have psychopathy but have not been charged with a violent crime.

As expected, such criticism sparked a heavy debate centred primarily on determining the “core” features of psychopathy. Sellbom and Drislane (25) provide a good overview of substantiated arguments in favour of meanness being the fundamental feature of psychopathy. However, it is essential to note that both their argumentation and the empirical evidence they provide support the meanness personality trait aspect, specifically the callous-unemotional (CU) traits, rather than the sense of personal empowerment associated with cruel behaviour towards others. This, in turn, indicates the possibility of individuals with high levels of psychopathic traits operating outside the forensic systems. Thus, the need to study this phenomenon in the general population remains.

To accommodate this, several self-report scales have been developed to assess psychopathy. Among these scales are the LSRP (7) TriPM (26), the Self-Report Psychopathy Scale-fourth edition (SRP-4; Paulhus et al. (27)), the Psychopathic Personality Traits Scale-Revised (PPTS-R); Budosek et al. (28), and the Psychopathic Processing and Personality Assessment (PAPA; Lewis et al. (29)). While these measures are more susceptible to biases associated with self-reported assessments than measures requiring collateral information, they nevertheless demonstrate adequate structure. They are related to the PCL-R (28–31).

In the Czech Republic, the predominant tool for diagnosing psychopathy is the International Classification of Diseases, Tenth Revision (ICD-10) (due to its codes being recognised by insurance companies). As such, the ICD-10 recognises only dissocial personality disorder (F60.2), and the Diagnostic and Statistical Manual of Mental Disorders-Fifth Edition (DSM-5) recognises only antisocial personality disorder (301.7). This may be one of the reasons why tools available in the Czech language that can capture psychopathy are lacking. The only internationally recognised tool translated into Czech is the PCL-R. However, no validation study has been published; consequently, the cutoff scores adjusted for the specific population are unknown. In other words, there are no validated tools for assessing or self-assessing psychopathy.

Consequently, there is a need for translated self-reported measures of psychopathy. Given that the PAPA has been shown to reflect potential sex differences in psychopathy, taps into all three elements of the TriPM, including CU traits, and is not centred on criminal behaviour (32), it was translated into Czech. This paper provides the results of the first validation of the translated PAPA. Therefore, this study has three main contributions. First, it allowed us to compare the conceptualisation of psychopathy in nonforensic samples between the United Kingdom (UK) and the Czech Republic, revealing the potential cultural impact on the construct. Second, the present study furthers the research on potential sex differences in psychopathy. Finally, the results contribute to the overall understanding of personality traits associated with psychopathy, thereby aiding in the question of its definition.

## Materials and methods

### Participants

The sample comprised 1013 community adults recruited via a data collection agency; 51.73% were male, and 48.27% were female. The average age of the sample was 42.14 years (SD = 8.03). All the participants had at least one child. Since the study aimed to validate the Czech translation of the PAPA, the total sample was randomly split (30:70; 304 and 709 participants, respectively) to conduct exploratory and confirmatory factor analyses.

### Study 1

The exploratory factor analysis included 304 participants (Mage = 42.12 years, SD = 8.31), of whom 50.33% were male, and 49.67% were female.

### Study 2

The confirmatory factor analysis included 709 participants (Mage = 42.15 years, SD = 7.91), of whom 52.33% were male, and 47.67% were female.

### Measures

All participants completed the PAPA (29), a self-report questionnaire assessing psychopathic traits. Data collection took place in the last week of May 2023. The PAPA comprises 29 items, each scored on a Likert-type scale. Participants rated whether the statement described them poorly (1: very unlike me) or accurately (5: very much like me). The PAPA includes four subscales: disregard for others, dissocial tendencies, responsivity to perceived aggression, and emotional detachment. Lewis et al. (32) reported that, in the community sample, the last subscale should be used only for female participants, not for male participants. The measure was first translated from English to Czech and then back-translated by another team member from Czech to English. Both translators were native Czech speakers proficient in English. The resulting differences were resolved via discussion between the research team and the scale’s author.

### Procedure

The STEM/MARK data collection agency conducted the data collection procedure. An online questionnaire was distributed via computer-assisted web interviewing (CAWI) methodology to collect the necessary data. The decision to employ an online survey was based on the high level of internet access among the adult population in the Czech Republic. The target respondents for this study were adult parents in the Czech Republic, as the questionnaire was part of a larger research project examining parental attitudes toward physical punishment. Notably, the sampling approach was nonrandom.

The inclusion criterion was that the participants were adult parents without any previous psychiatric disorders. To ensure a diverse and representative sample, quota sampling was employed, ensuring that the sample composition with respect to age, sex, education level, and geographic region matched that of the general Czech population. Before participation, informed consent was obtained online, and the ethical committee of the General Faculty Hospital of Charles University, Prague, approved the research protocol.

### Data analysis

All the data were analysed via R software. Owing to differences in PAPA structure across forensic and community samples, as well as between male and female participants, a principal component analysis was performed. After the PAPA structure in the Czech community sample was examined, confirmatory factor analysis was conducted to verify the structure and to investigate potential differences between male and female participants.

## Results

The correlation matrix did not indicate any problems: despite significant associations among items, no correlation coefficient exceeded.53, suggesting no duplicate items. The overall Kaiser Meyer Olkin value of.88 and statistically significant Bartlett test indicated that the data is suitable for exploratory factor analysis. Parallel Analysis using Horn’s method (33) recommended a factor structure. To verify the recommended number of factors, CFI- and RAMSEA-based hull methods were implemented in FACTOR (34, 35). The latter recommended a unidimensional structure as most optimal. Although this was in stark divergence from the theoretical models of psychopathy and original scale structure, we have explored this avenue. The resulting PCA with varimax rotation (using the same parameters as Lewis et al., 2021) showed that items 5, 9, 16, 18, 24, and 26 loaded below.40 and were removed for subsequent confirmatory factor analysis. However, once the single factor solution was fitted against the second part of the data, it demonstrated poor model fit, even with specified constraints (for males CFI: 0.88, RMSEA:0.07, SRMR: 0.05, GFI: 0.87, and ECVI: 1.71, and for females CFI: 0.85, RMSEA:0.08, SRMR: 0.06, GFI: 0.86, and ECVI: 1.93). Consequently, because the one-factor solution failed to adequately capture theoretically meaningful distinctions consistently reported in prior literature and demonstrated poor fit with the data, we opted to follow up with the three- and four-factor solutions suggested as most optimal by the parallel analysis. This decision was made with the understanding that dimensionality heuristics should be interpreted in conjunction with model fit, theoretical coherence, and construct validity, rather than based solely on indices.

Principal component analysis (PCA) with varimax rotation was conducted for four- and three-factor structures. The results are presented in **Tables 1** and **2**. Interestingly, the four-factor solution had poor correspondence with the structure identified by Lewis et al. (32). In addition to the items that did not load on any factor other than Item 23,^2^ Items 6, 11, 24, and 26 were compared between our study and that by Lewis et al. (32), but the factor structures did not match. The most significant overlap between the solution identified in this analysis and that determined by Lewis et al. (32) occurred for four items in the disregard factor: Items 1, 2, 17, and 20. However, the present study also identified four additional items. Owing to disparities in the factor structure, the analysis was guided by theoretical considerations and data rather than by Lewis et al.’s (32) study.

**Table 1:** PAPA loading with four factors.

Item	Factor 1	Factor 2	Factor 3	Factor 4
papa_28	0.676			
papa_12	0.632			
papa_13	0.616			
papa_29	0.575			
papa_21	0.563			
papa_19	0.552			
papa_27	0.497			
papa_7	0.491			
papa_14	0.403			
papa_11	0.401			
papa_23				
papa_2		0.629		
papa_20		0.585		
papa_6		0.584		
papa_1		0.539		
papa_25		0.527		
papa_3		0.516		
papa_17		0.457		
papa_22		0.436		
papa_4				
papa_5				
papa_26			0.696	
papa_16			0.692	
papa_24			0.646	
papa_18			0.611	
papa_9				0.719
papa_8				0.623
papa_10		0.507		0.51
papa_15				0.423

The item key is presented in the **Appendix A**.

**Table 2:** PAPA loading with 3 factors.

Item	Factor 1	Factor 2	Factor 3
papa_28	0.657		
papa_13	0.62		
papa_12	0.606		
papa_21	0.579		
papa_27	0.573		
papa_7	0.543		
papa_29	0.54		
papa_8	0.504		
papa_14	0.483		
papa_15	0.482		
papa_19	0.463		
papa_23			
papa_11			
papa_2		0.643	
papa_20		0.604	
papa_6		0.598	
papa_1		0.556	
papa_25		0.54	
papa_10	0.434	0.532	
papa_3		0.526	
papa_17		0.468	
papa_22		0.434	
papa_4			
papa_9			
papa_5			
papa_26			0.712
papa_16			0.693
papa_24			0.651
papa_18			0.579

The item key is presented in the **Appendix A**.

The first factors in both of the yielded solutions matched in terms of Item 7 (Others would describe me as an irritable person who has difficulty controlling their emotions), Item 12 (I get into trouble often and more than others), Item 13 (I find it hard to comfort others when they are upset), Item 14 (I do not care about other people), Item 19 (I am capable of behaving in such a way that my behaviour can get me into trouble), Item 21 (I am an aggressive person in many situations), Item 27 (I often find people to be aggressive or hostile towards me), Item 28 (Others would describe me as a very pushy person who has trouble winning over others), and Item 29 (As a child, I got into trouble more often than others. The only item from the four-factor solution that did not load on this factor in the three-factor solution was Item 11 (I find it impossible to resist temptation), which did not load on any factor. However, the loading in the four-factor solution was borderline (.40), suggesting that it did not adequately capture the underlying construct. Given that the items with the highest loadings on this factor were Items 28, 13, and 12, which represent hostile attitudes toward others, the absence of an item related to impulse control from this factor is reasonable.

Furthermore, in the three-factor solution, this factor included three out of four items loading on the fourth factor in the four-factor solution, specifically, Item 8 (I see much hostility around me), Item 10 (In my opinion, most people are weak and not worth being bothered with), and Item 15 (The world is a dangerous place, and you need to “cover your back”). Since Items 8 and 15 are directly related to hostile attitudes toward others, retaining these items, along with the three-factor solution, resulted in a more economical structure. The first component addresses the hostility aspect of psychopathy.

The second factor in the three-factor solution included all of the items from the second factor in the four-factor solution. These included items describing self-centeredness and manipulative attitudes towards others, specifically Item 1 (I find interest only in myself), Item 2 (I use people to obtain what I want), Item 3 (I usually choose the riskier option, either for me or for others), Item 6 (Others describe me as a cruel person who has no fear of harming others), Item 17 (If I am caught in a lie, I can quickly figure out how to get out of it), Item 20 (It often happens that I see others only as “objects” or tools to be used), Item 22 (I use illegal drugs or medicines that are not meant for me, more so than other people I know), and Item 25 (I often think that I am more critical than others). Although Item 10 was the only item in the three-factor solution that loaded on more than one factor, more muscular loading (.53 vs.43) and a theoretical relation to manipulation rather than hostility firmly placed it in this factor, which underpins manipulativeness.

The last factor in the three-factor solution included only four items: Item 16 (I often empathize with other people’s feelings), Item 18 (I often experience strong positive emotions such as feelings of happiness and joy), Item 24 (When I do something wrong, I feel bad), and Item 26 (I always accept responsibility for what I do). All of these items are reverse-scored, represent the CU traits, and correspond to the third factor in the four-factor solution. Thus, given both the theoretical explanations and the simplicity of the three-factor structure, this factor was retained for subsequent analysis.

First, the internal consistency of the subscales identified in Study 1 was verified for each sex. The Cronbach’s alphas for all scales ranged from acceptable to good, indicating suitable internal consistency of the subscales: Factor 1, 0.86 for men and 0.85 for women; Factor 2, 0.84 for men and 0.85 for women; and Factor 3, 0.64 for men and 0.68 for women.

Subsequently, separate confirmatory analyses with free parameters were conducted for men and women using the three-factor solutions identified in Study 1. The initial fit for males was borderline acceptable (comparative fit index (CFI): 0.82, root mean square error of approximation (RMSEA): 0.08, standardised root mean squared residual (SRMR): 0.07, goodness-of-fit index (GFI): 0.84, and expected cross-validation index (ECVI): 2.5). Similarly, for the female participants, the model did not fit correctly (CFI: 0.84, RMSEA: 0.07, SRMR: 0.06, GFI: 0.85, and ECVI: 2.36). Consequently, both models were refined by evaluating poor loadings and specific covariances. Items 15 (.41 for males and.48 for females), 17 (.37 for males and.40 for females), and 18 (.30 for males and.36 for females) had loadings below 0.50 for both sexes; therefore, they were removed. The following reasoning augmented this decision. However, Items 8 (.47) and 14 (.49) had low loadings only in the male sample. Therefore, to test the validity of psychopathy assessment using the same instrument in males and females, it was decided to retain these two items for the male sample. Furthermore, while Item 15 is the only item in the hostility subscale that implies the necessity to protect oneself (the need to “cover your back”), which suggests the possibility of the reactive nature of one’s behaviour, Items 8 and 14 focus on an individual’s hostile attitudes towards others.

**Figure 1:**
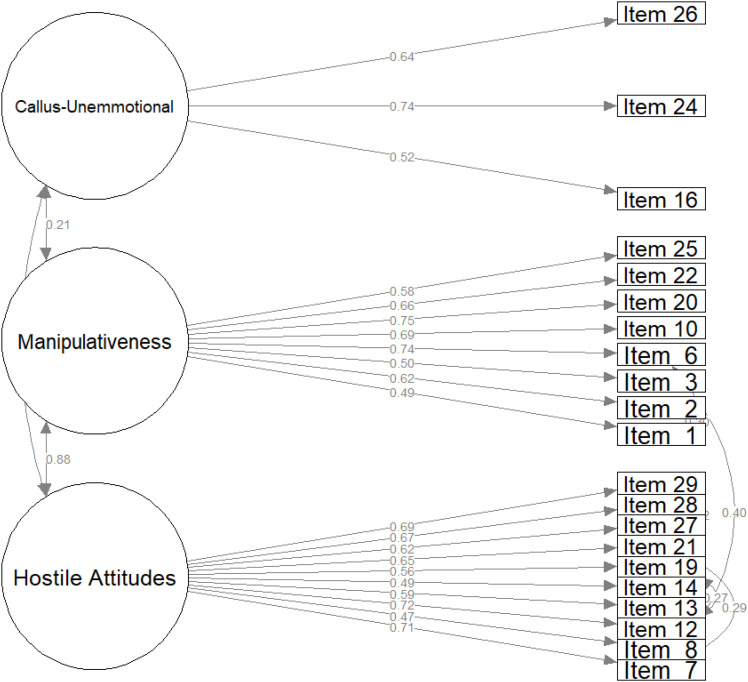
Confirmatory factor analysis of PAPA structure in male sample.

**Figure 2:**
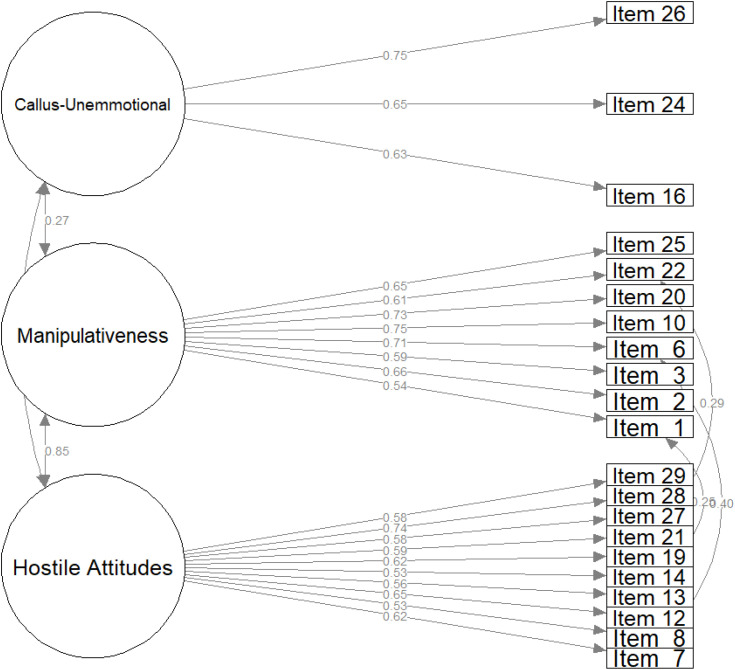
Confirmatory factor analysis of PAPA structure in the male sample.

As a result, the model fit for both males (CFI: 0.86, RMSEA: 0.08, SRMR: 0.06, GFI: 0.87, and ECVI: 1.82) and females (CFI: 0.86, RMSEA: 0.08, SRMR: 0.06, GFI: 0.87, and ECVI: 1.89) improved. In the next step, the covariances were specified using the covariance matrix. Interestingly, Items 7 and 6, which belong to different subscales, shared residual covariance in both the male and female samples. Moreover, Items 7 and 21, 12 and 19, and 27 and 28 belonged to the hostility subscale, and Items 1 and 2 belonged to the manipulativeness subscale, with only shared covariance in the male sample. However, cross-subscale shared variance was more prevalent in the female sample, specifically among Items 14, 1, 21, and 22. The resulting models showed good fit for both the male (CFI: 0.92, RMSEA: 0.06, SRMR: 0.05, GFI: 0.9, and ECVI: 1.41) and female (CFI: 0.9, RMSEA: 0.07, SRMR: 0.05, GFI: 0.89, and ECVI: 1.63) samples, as shown in **Figures 1** and **2**. To verify the decision to retain Items 8 and 14 in the male sample, we tested the final model without them (CFI: 0.93, RMSEA: 0.06, SRMR: 0.05, GFI: 0.92, and ECVI: 1.41). Nevertheless, as the changes in fit were minute, these items needed to be retained. As a result, our final factor solutions showed good fits for both male and female participants. Finally, model fit for the factor structures reported by Lewis et al. (32) was estimated, with a CFI of 0.86, an RMSEA of 0.08, an SRMR of 0.09, a GFI of 0.87, and an ECVI of 1.61 for the male sample and a CFI of 0.82, an RMSEA of 0.08, an SRMR of 0.1, a GFI of 0.84, and an ECVI of 2.36 for the female sample, with these models having a poorer fit.

## Discussion

This is the first study validating the PAPA for use in the Czech Republic. The results showed that, although the scale fit the sample of male and female parents, the structure identified differed significantly from those identified by Lewis et al. (29, 32). First, the data from the present study suggested a three-factor solution suitable for both male and female participants, in contrast to the three- and four-factor solutions reported by Lewis et al. (32) for males and females, respectively. Second, the three-factor solution identified in this study comprised only 21 items, compared with the 19-item solution for males and the 23-item solution for females reported for the UK population. Finally, the composition of the three factors in this study differed from that in previous research with respect to gender composition.

The divergence from the factor composition reported by Lewis et al. (32) was evident in the PCA results. Although parallel analysis identified three- and four-factor structures, neither structure resembled those from the previous studies. The four-factor solution likely performed poorly due to restricted variance and low base rates of severe psychopathic traits in a community sample. The lower Cronbach’s alpha (<.70) for the callous-unemotional factor reinforces this supposition. Furthermore, in non-forensic samples, lower levels of psychopathy-related traits might overlap to form continuous dimensions rather than independent factors, reinforcing the utility of dimensional approaches to assessment. Therefore, this difference may be attributable to the use of separate PCAs for males and females in the study by Lewis et al. (32). However, given that there are concerns regarding the differences in psychopathy presentations assessed by existing instruments (19–21), the present study attempted to explore a structural fit for both men and women. Moreover, in the original validation study, Lewis et al. (29) performed PCA on a sample comprising both sexes. Although this sample comprised prisoners, the rigid structure would have left at least some overlap in the core items between forensic and nonforensic samples. Given that this was not the case, the present study provides a basis for a new, refined structure of the PAPA among participants in the Czech Republic.

The two significant benefits of the structure identified by confirmatory factor analysis in this study are that it reduces the number of scale items by eight and yields the same structure for men and women. In addition, the three-factor model had acceptable CFI, RMSEA, and SRMR values, indicating a good fit. Furthermore, all fit indices for both sexes indicated better fit for the three-factor solution than the solutions reported by Lewis et al. (32), with the only exception being the CFI for the female sample, which was slightly lower in the present study. Although it is impossible to claim that the proposed three-factor solution outperforms the previous solutions, given the marginal size of the differences, this solution still fits the data equally well, thereby presenting an alternative structure for the PAPA in the general population. However, the clear advantage of the three-factor model over previous models in the present study is that it is better suited to the general population in the Czech Republic. This makes the PAPA the only validated assessment of psychopathy in the Czech Republic.

The three-factor model proposed in this study includes hostile attitudes, manipulativeness, and CU traits as core aspects of psychopathy. While these factors do not precisely align with the two overarching factors or the four subfactors of the PCL-R (2, 16, 35), there are more similarities than differences. Like the PCL-R, the PAPA incorporates factors reflecting affective and interpersonal domains, including CU traits and manipulativeness. Unlike the PCL-R, however, the PAPA does not include criminal versatility or criminal behaviour as indicators of psychopathy, thereby preventing the circular definition problem associated with the PCL-R (12). Moreover, the hostile attitudes factor of the PAPA partially matches the lifestyle facet of the PCL-R, specifically concerning poor behavioural control. For example, consider the item “Others would describe me as an irritable person who has difficulty controlling their emotions”.

Similarly, the three-factor solution partially agrees with the TriPM (22). The hostile attitudes factor overlaps with the *disinhibition* aspect of the TriPM, as the former includes items describing externalising behaviour. Moreover, both the manipulativeness and CU trait factors appear to correspond to the *meanness* factor. While the latter factor from the current model reflects cold-heartedness, the former corresponds to empowerment through cruelty to others. This means that the current three-factor structure of the PAPA provides an in-depth assessment of the “core” aspect of psychopathy (25).

Nevertheless, similar to the PCL-R, the PAPA should draw on the triarchic model of psychopathy and focus more on the *boldness* dimension. Given that it is suggested to be a potentially adaptive aspect of psychopathy (23, 24), it should be captured by a self-report tool designed for the general population and the forensic population. However, the original PAPA lacked items related to this aspect of psychopathy (32). Although it is not feasible to add items in a validation study, given the reduction in the number of items in the proposed solution, adding a new factor is a promising avenue for future research on this scale.

There are further limitations to this study. First, owing to the lack of a validated measure of psychopathy in the Czech Republic, it was impossible to assess the diagnostic accuracy or convergent validity against an established measure of psychopathy. Second, the present study relied exclusively on a sample of adults with at least one child. Thus, while there is potential for applying the current structure to a broader population, further research is needed; its application to community populations remains tentative, and its application to forensic populations remains hypothetical. Third, given the need to perform both exploratory and confirmatory factor analyses on the sample, the present study included the minimal required sample size. A larger sample size would provide more robust results. Lastly, although Item Response Theory offers powerful tools for item-level analysis, it was not employed in the current study. The objective of this research was to replicate and evaluate the factor structure of the original instrument in the context of translation and cultural adaptation. As the original development and validation were conducted using Classical Test Theory, the same framework was retained to preserve methodological continuity.

Nevertheless, the primary goal of the present study was to validate the structure of the Czech version of the PAPA. This finding shows that personality functioning associated with psychopathy can be assessed cross-culturally. However, the cultural change also resulted in differences in the scale’s structure. The two main reasons for such changes are either poor stability of the initial factor structure or the pervasive influence of culture. Both possibilities should be verified in future research using the newly proposed three-factor PAPA structure.

Furthermore, until further studies using forensic samples are conducted, the current results have only preliminary and theoretical implications for this population. Moreover, there is still a lack of assessment of the psychopathy traits associated with the ability to stay calm in pressing situations. Importantly, this free-factor structure was similar in males and females, showing that in nonforensic populations, psychopathy traits might manifest similarly between men and women.

## Data Availability

The raw data supporting the conclusions of this article will be made available by the authors, without undue reservation.

## Footnotes

1 The triarchic model of psychopathy refers to a theoretical framework describing three core dimensions (boldness, meanness, and disinhibition), whereas the Triarchic Psychopathy Measure represents one specific operationalisation of this framework. The instrument examined in the present study targets the same conceptual dimensions, but constitutes an independent operationalisation rather than a direct adaptation of the Triarchic Psychopathy Measure.

2 All items are presented in **Appendix A**.
