# Supplementary material for: Validation of the Psychopathic Processing and Personality Assessment in the Czech Republic
Source: Front Psychiatry. 2026 Feb 20;17:1694620. doi: 10.3389/fpsyt.2026.1694620 (PMC12964200; doi:10.3389/fpsyt.2026.1694620)
Supplement: Supplementary file 1 [file Table1.docx]

Appendix A

PAPA items key

1. I only find interest in myself.

2. I use people to get what I want.

3. I usually choose the option that is more risky, either for me or for others.

4. I usually don't think about the consequences of my behaviour.

5. Personally, I never change.

6. They describe me as a cruel person who has no fear of harming others.

7. Others would describe me as an irritable person who has difficulty controlling his emotions.

8. I see a lot of hostility around me.

9. People around me seem lazy.

10. In my opinion, most people are weak and not worth being bothered with.

11. I find it impossible to resist temptation.

12. I get into trouble often and more than others.

13. I find it hard to comfort others when they are upset.

14. I don't care about other people.

15. The world is a dangerous place and you need to "cover your back".

16. I often empathize with other people's feelings.

17. If I'm caught in a lie, I can quickly figure out how to get out of it.

18. I often experience strong positive emotions such as feelings of happiness and joy.

19. I am capable of behaving in such a way that my behavior can get me into trouble.

20. It often happens that I see others only as "objects" or tools to be used.

21. I am an aggressive person in many situations.

22. I use illegal drugs or medicines that are not meant for me, more than other people I know.

23. I find it difficult to give emotional or personal support to others.

24. When I do something wrong, I feel bad.

25. I often think that I am more important than others.

26. I always accept responsibility for what I do.

27. I often find people to be aggressive or hostile towards me.

28. Others would describe me as a very pushy person who has trouble winning over others.

29. As a child, I got into trouble more often than others.
